# 1-Furoyl-3-[3-(trifluoro­meth­yl)phen­yl]thio­urea

**DOI:** 10.1107/S1600536809013038

**Published:** 2009-04-10

**Authors:** Jahyr E. Theodoro, O. Estévez-Hernández, J. Ellena, J. Duque, Rodrigo S. Corrêa

**Affiliations:** a Grupo de Cristalografía, Instituto de Física de São Carlos, Universidade de São Paulo, São Carlos, Brazil; b Laboratory of Molecular Ingeniery, Institute of Materials, University of Havana, Cuba

## Abstract

The title compound, C_13_H_9_F_3_N_2_O_2_S, crystallizes with two independent mol­ecules in the asymmetric unit. The central thio­urea core is roughly coplanar with the furan and benzene rings, showing O—C—N—C(S) torsion angles of 2.3 (4) and −11.4 (2)° and (S)C—N—C—C torsion angles of −2.4 (4) and −28.8 (4)°, respectively, in the two independent mol­ecules. The *trans*–*cis* geometry of the thio­urea fragment is stabilized by an intra­molecular N—H⋯O hydrogen bond between the H atom of the *cis* thio­amide and the carbonyl O atom. In the crystal structure, inter­molecular N—H⋯S hydrogen bonds form centrosymmetric dimers extending along the *b* axis.

## Related literature

For general background to aroylthio­ureas, see: Aly *et al.* (2007 ▶); Koch (2001 ▶); Estévez-Hernández *et al.* (2007 ▶); Otazo-Sánchez *et al.* (2002 ▶). For related structures, see: Theodoro *et al.* (2008 ▶); Pérez *et al.* (2008 ▶). For the synthesis, see: Otazo-Sánchez *et al.* (2001 ▶).
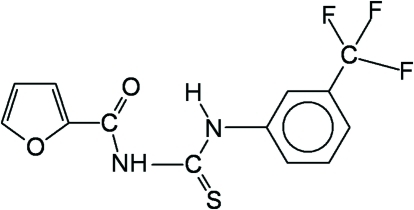


## Experimental

### 

#### Crystal data

C_13_H_9_F_3_N_2_O_2_S
*M* *_r_* = 314.29
Triclinic, 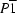

*a* = 7.5540 (14) Å
*b* = 13.684 (5) Å
*c* = 14.210 (3) Å
α = 86.124 (13)°
β = 74.779 (7)°
γ = 74.065 (8)°
*V* = 1362.9 (6) Å^3^
*Z* = 4
Mo *K*α radiation
μ = 0.28 mm^−1^
*T* = 294 K
0.09 × 0.07 × 0.03 mm

#### Data collection

Enraf–Nonius KappaCCD diffractometer
Absorption correction: none
9271 measured reflections
4953 independent reflections
2925 reflections with *I* > 2σ(*I*)
*R*_int_ = 0.035

#### Refinement

*R*[*F* ^2^ > 2σ(*F* ^2^)] = 0.044
*wR*(*F* ^2^) = 0.123
*S* = 1.01
4953 reflections
433 parameters
H-atom parameters constrained
Δρ_max_ = 0.14 e Å^−3^
Δρ_min_ = −0.24 e Å^−3^

### 

Data collection: *COLLECT* (Nonius, 2000 ▶); cell refinement: *SCALEPACK* (Otwinowski & Minor, 1997 ▶); data reduction: *DENZO* (Otwinowski & Minor, 1997 ▶) and *SCALEPACK*; program(s) used to solve structure: *SHELXS97* (Sheldrick, 2008 ▶); program(s) used to refine structure: *SHELXL97* (Sheldrick, 2008 ▶); molecular graphics: *ORTEP-3 for Windows* (Farrugia, 1997 ▶) and *Mercury* (Macrae *et al.*, 2006 ▶); software used to prepare material for publication: *WinGX* (Farrugia, 1999 ▶).

## Supplementary Material

Crystal structure: contains datablocks global, I. DOI: 10.1107/S1600536809013038/fj2202sup1.cif

Structure factors: contains datablocks I. DOI: 10.1107/S1600536809013038/fj2202Isup2.hkl

Additional supplementary materials:  crystallographic information; 3D view; checkCIF report

## Figures and Tables

**Table 1:** Hydrogen-bond geometry (Å, °)

*D*—H⋯*A*	*D*—H	H⋯*A*	*D*⋯*A*	*D*—H⋯*A*
N1—H1⋯S1*A*^i^	0.86	2.78	3.643 (2)	176
N1—H1⋯O2	0.86	2.27	2.692 (3)	110
N1*A*—H1*A*⋯S1^ii^	0.86	2.74	3.592 (2)	173
N1*A*—H1*A*⋯O2*A*	0.86	2.30	2.700 (3)	108
N2—H2⋯O1	0.86	1.88	2.625 (3)	144
N2*A*—H2*A*⋯O1*A*	0.86	1.92	2.640 (3)	140

Symmetry codes: (i) 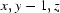; (ii) 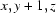.

**Fig. 1.**
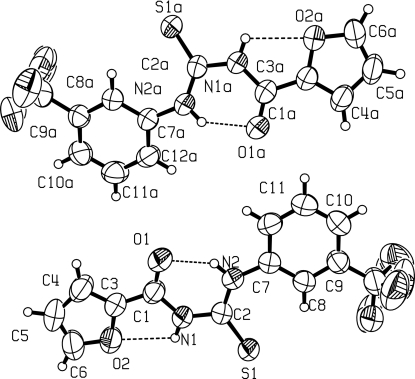
The molecular structure of the title compound. Displacement ellipsoids are drawn at the 50% probability level. The intramolecular N—H···O hydrogen bond is shown as a dashed line. For clarity reason the atoms (C13, C13a, F1, F1a, F2, F2a, F3 and F3a) of the trifluoromethyl groups are not labeled.

**Fig. 2.**
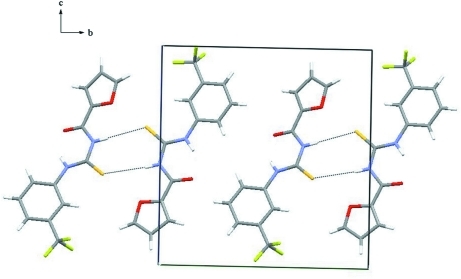
View of the crystal packing of the title compound. Intermolecular hydrogen bonds are shown as dashed lines.

## supplementary crystallographic information

### Comment

The importance of aroylthioureas is found largely in heterocyclic syntheses and many of these substrates have interesting biological activities. Aroylthioureas have also been found to have applications in metal complexes and molecular electronics (Aly *et al.*, 2007). The title compound (I), Fig. 1, was synthesized from furoyl isothiocyanate and 3-trifluorometylaniline in dry acetone. This thiourea derivative has been successfully used as ionophore in amperometric sensor for Cd(II) (Estévez-Hernández *et al.*, 2007). The title compound crystallizes in the thioamide form with two independent molecules in the asymmetric unit. The main bond lengths are within the ranges obtained for similar compounds (Koch *et al.*, 2001 and Pérez *et al.*, 2008). The C2—S1 and C1—O1 bonds (Table 1) both show the expected double-bond character. The short values of the C2—N1, C2—N2 and C1—N2 bonds indicate partial double bond character. These results can be explained by the existence of resonance in this part of the molecule. The C=S distance for compound I (two unique molecules) averages 1.648 Å. The furan carbonyl (O1—C1—C3—O2 and O1a—C1a—C3a—O2a, two unique molecules) groups are inclined 2.3 (4)° and -11.4 (2)° with respect to the plane formed by the thiourea moiety (N1—C2—S1—N2 and N1a—C2a—S2—N2a, two unique molecules)in each molecule, while the 3-trifluoromethylphenyl (C7—C8—C9—C10—C11 and C7a—C8a—C9a—C10*a*—C11*a*, two unique molecules) rings are inclined -2.4 (4)° and -28.8 (4)°, respectively. In addition, the dihedral angles of two independent molecules between the furan and benzene ring planes are 18.91 (1)° and 14.78 (1)°, respectively. The *trans*-*cis* geometry in the thiourea moiety is stabilized by the N2—H2···.O1 intramolecular hydrogen bond. This strong interaction is also observed in solution (Otazo-Sánchez *et al.*, 2002) and locks the –CONHCSNHR– unit into a stable planar six-membered ring structure (Fig.1 and Table 1). In this S-shaped conformation between the C=O and C=S groups (two donors sites rich in electron density), the O—S distance is maximum, contributing to a minimum conformational energy of the molecule as a whole (Koch *et al.*, 2001). Another weaker intramolecular hydrogen interaction between the furan oxygen atom O2 and the N1—H1 hydrogen atom is observed. The crystal structure is stabilized by two intermolecular N1—H1···.S1 hydrogen bonds (Fig.2 and Table 1) between related molecules forming dimers pilled within the unit cell along the [010] direction.

### Experimental

The title compound (I) was synthesized according to a previous report (Otazo-Sánchez *et al.*, 2001), by converting furoyl chloride into furoyl isothiocyanate and then condensing with 3-trifluorometylaniline. The resulting solid product was crystallized from ethanol yielding X-ray quality single crystals (m.p 112–113 °C). Elemental analysis (%) for C_13_H_9_N_2_O_2_F_3_S calculated: C 49.68, H 2.87, N 8.92, S 10.19; found: C 49.46, H 2.86, N 15.79, S 10.09.

### Refinement

All H atoms were refined with *U*_iso_(H)=1.2*U*_eq_(C/N).

A disordered behavior was observed for the fluorine atoms. Their anisotropic thermal parameters are particularly high, for F1, F2 and F3, respectively. However they are too far from the thiourea core to induce any effect on its nucleophilic centers. The position that these fluorine atoms occupy, at the end of the molecule, favors the behavior observed. The C13A and C13 atoms of the trifluoromethyl groups also show a high anisotropic thermal parameter. This appears an effect induced by the fluorine atoms movement.

### Crystal data

**Table:**

C_13_H_9_F_3_N_2_O_2_S	*Z* = 4
*M**_r_* = 314.29	*F*(000) = 640
Triclinic, *P*1	*D*_x_ = 1.532 Mg m^−^^3^
Hall symbol: -P 1	Mo *K*α radiation, λ = 0.71073 Å
*a* = 7.5540 (14) Å	Cell parameters from 8001 reflections
*b* = 13.684 (5) Å	θ = 2.9–26.7°
*c* = 14.210 (3) Å	µ = 0.28 mm^−^^1^
α = 86.124 (13)°	*T* = 294 K
β = 74.779 (7)°	Prism, colourless
γ = 74.065 (8)°	0.09 × 0.07 × 0.03 mm
*V* = 1362.9 (6) Å^3^	

### Data collection

**Table:**

Enraf–Nonius KappaCCD diffractometer	2925 reflections with *I* > 2σ(*I*)
Radiation source: fine-focus sealed tube Enraf Nonius FR590	*R*_int_ = 0.035
horizontally mounted graphite crystal	θ_max_ = 25.4°, θ_min_ = 2.9°
φ scans and ω scans winth κ offsets	*h* = −9→9
9271 measured reflections	*k* = −16→16
4953 independent reflections	*l* = −17→17

### Refinement

**Table:**

Refinement on *F*^2^	0 restraints
Least-squares matrix: full	H-atom parameters constrained
*R*[*F*^2^ > 2σ(*F*^2^)] = 0.044	*w* = 1/[σ^2^(*F*_o_^2^) + (0.058*P*)^2^ + 0.0881*P*] where *P* = (*F*_o_^2^ + 2*F*_c_^2^)/3
*wR*(*F*^2^) = 0.123	(Δ/σ)_max_ < 0.001
*S* = 1.01	Δρ_max_ = 0.14 e Å^−^^3^
4953 reflections	Δρ_min_ = −0.24 e Å^−^^3^
433 parameters	

### Special details

**Table:**

Geometry. All e.s.d.'s (except the e.s.d. in the dihedral angle between two l.s. planes) are estimated using the full covariance matrix. The cell e.s.d.'s are taken into account individually in the estimation of e.s.d.'s in distances, angles and torsion angles; correlations between e.s.d.'s in cell parameters are only used when they are defined by crystal symmetry. An approximate (isotropic) treatment of cell e.s.d.'s is used for estimating e.s.d.'s involving l.s. planes.

### Fractional atomic coordinates and isotropic or equivalent isotropic displacement parameters (Å^2^)

**Table:**

	*x*	*y*	*z*	*U*_iso_*/*U*_eq_	Occ. (<1)
C1	0.3542 (4)	0.0592 (2)	0.38462 (18)	0.0710 (7)	
C2	0.1946 (3)	0.02259 (17)	0.55546 (17)	0.0645 (6)	
C3	0.4502 (4)	0.01038 (19)	0.29077 (17)	0.0710 (7)	
C4	0.5248 (4)	0.0463 (2)	0.2042 (2)	0.0936 (9)	
H4	0.5281	0.1131	0.1895	0.112*	
C5	0.5978 (5)	−0.0373 (3)	0.1393 (2)	0.1019 (10)	
H5	0.6575	−0.036	0.0733	0.122*	
C6	0.5654 (5)	−0.1173 (3)	0.1899 (2)	0.1062 (10)	
H6	0.5997	−0.1828	0.1647	0.127*	
C7	0.0783 (3)	0.17521 (17)	0.66537 (17)	0.0655 (6)	
C8	−0.0076 (4)	0.13680 (18)	0.75310 (17)	0.0712 (6)	
H8	−0.0151	0.0699	0.7569	0.085*	
C9	−0.0816 (4)	0.19813 (18)	0.83429 (18)	0.0707 (6)	
C10	−0.0735 (4)	0.2979 (2)	0.8295 (2)	0.0869 (8)	
H10	−0.1238	0.339	0.8849	0.104*	
C11	0.0087 (4)	0.3353 (2)	0.7432 (2)	0.0930 (9)	
H11	0.0132	0.4028	0.7395	0.112*	
C12	0.0852 (4)	0.27506 (19)	0.6615 (2)	0.0782 (7)	
H12	0.1422	0.3017	0.6031	0.094*	
C13	−0.1660 (6)	0.1558 (3)	0.9296 (2)	0.0923 (9)	
O1	0.3279 (3)	0.15065 (15)	0.39493 (13)	0.0941 (6)	
O2	0.4751 (3)	−0.09106 (15)	0.28394 (13)	0.0929 (6)	
N1	0.2953 (3)	−0.00303 (14)	0.45947 (13)	0.0678 (5)	
H1	0.3248	−0.0665	0.445	0.081*	
N2	0.1654 (3)	0.11989 (15)	0.57768 (15)	0.0745 (6)	
H2	0.2079	0.1556	0.5293	0.089*	
S1	0.12519 (11)	−0.06681 (5)	0.62741 (5)	0.0817 (2)	
F2	−0.3379 (18)	0.2058 (8)	0.9735 (10)	0.145 (5)	0.6
F1	−0.0713 (15)	0.1517 (11)	0.9950 (9)	0.138 (4)	0.6
F3	−0.1806 (18)	0.0624 (6)	0.9246 (6)	0.135 (4)	0.6
F11	−0.121 (4)	0.1867 (19)	1.0000 (13)	0.206 (11)	0.4
F21	−0.356 (3)	0.1899 (16)	0.9474 (15)	0.158 (8)	0.4
F31	−0.125 (3)	0.0578 (8)	0.9282 (11)	0.161 (7)	0.4
C1A	0.0472 (3)	0.6271 (2)	0.62271 (17)	0.0678 (6)	
C2A	0.2779 (3)	0.65003 (18)	0.46698 (16)	0.0631 (6)	
C3A	−0.0434 (3)	0.67263 (18)	0.71851 (17)	0.0677 (6)	
C4A	−0.1499 (4)	0.6405 (2)	0.79957 (19)	0.0834 (8)	
H4A	−0.1881	0.5808	0.8067	0.1*	
C5A	−0.1920 (4)	0.7148 (2)	0.8713 (2)	0.0939 (9)	
H5A	−0.2637	0.7136	0.9353	0.113*	
C6A	−0.1105 (4)	0.7871 (2)	0.8307 (2)	0.0924 (9)	
H6A	−0.1161	0.8454	0.8626	0.111*	
C7A	0.4030 (3)	0.49953 (18)	0.35601 (16)	0.0629 (6)	
C8A	0.4819 (3)	0.54077 (18)	0.26840 (17)	0.0693 (6)	
H8A	0.4581	0.6109	0.2612	0.083*	
C9A	0.5957 (3)	0.47733 (19)	0.19206 (17)	0.0673 (6)	
C10A	0.6284 (4)	0.3734 (2)	0.2009 (2)	0.0793 (7)	
H10A	0.7044	0.3309	0.149	0.095*	
C11A	0.5474 (4)	0.3332 (2)	0.2875 (2)	0.0841 (8)	
H11A	0.5688	0.2632	0.2941	0.101*	
C12A	0.4355 (4)	0.39553 (18)	0.36399 (19)	0.0724 (7)	
H12A	0.3807	0.3674	0.422	0.087*	
C13A	0.6833 (5)	0.5235 (3)	0.1000 (2)	0.0856 (8)	
O1A	0.0331 (3)	0.54417 (14)	0.60375 (13)	0.0879 (6)	
O2A	−0.0178 (2)	0.76400 (13)	0.73597 (12)	0.0789 (5)	
N1A	0.1507 (3)	0.68170 (14)	0.55649 (13)	0.0672 (5)	
H1A	0.1345	0.7436	0.5726	0.081*	
N2A	0.2842 (3)	0.55799 (14)	0.43803 (13)	0.0693 (5)	
H2A	0.2028	0.5299	0.4754	0.083*	
S1A	0.40615 (12)	0.72621 (6)	0.40944 (5)	0.0884 (3)	
F2A	0.5546 (12)	0.5748 (9)	0.0533 (6)	0.136 (3)	0.6
F3A	0.7983 (13)	0.4580 (9)	0.0347 (7)	0.137 (5)	0.6
F1A	0.7723 (19)	0.5875 (10)	0.1111 (4)	0.138 (4)	0.6
F2B	0.594 (3)	0.6154 (10)	0.0881 (10)	0.157 (6)	0.4
F1B	0.8549 (18)	0.5299 (15)	0.1025 (12)	0.171 (8)	0.4
F3B	0.710 (4)	0.4729 (16)	0.0251 (10)	0.193 (9)	0.4

### Atomic displacement parameters (Å^2^)

**Table:**

	*U*^11^	*U*^22^	*U*^33^	*U*^12^	*U*^13^	*U*^23^
C1	0.0753 (17)	0.0767 (17)	0.0623 (16)	−0.0278 (13)	−0.0144 (13)	0.0109 (13)
C2	0.0713 (15)	0.0653 (15)	0.0570 (14)	−0.0215 (12)	−0.0131 (12)	0.0013 (11)
C3	0.0786 (17)	0.0744 (17)	0.0596 (15)	−0.0261 (13)	−0.0130 (13)	0.0086 (12)
C4	0.103 (2)	0.098 (2)	0.0745 (19)	−0.0383 (17)	−0.0082 (16)	0.0213 (17)
C5	0.107 (2)	0.133 (3)	0.0561 (17)	−0.032 (2)	−0.0066 (16)	0.0116 (19)
C6	0.128 (3)	0.109 (2)	0.0667 (19)	−0.027 (2)	−0.0025 (18)	−0.0139 (18)
C7	0.0716 (16)	0.0600 (14)	0.0641 (15)	−0.0196 (12)	−0.0131 (12)	−0.0012 (11)
C8	0.0893 (18)	0.0601 (14)	0.0639 (15)	−0.0234 (13)	−0.0149 (13)	−0.0012 (12)
C9	0.0757 (17)	0.0664 (16)	0.0665 (16)	−0.0149 (12)	−0.0150 (13)	−0.0047 (12)
C10	0.098 (2)	0.0719 (18)	0.082 (2)	−0.0179 (15)	−0.0083 (16)	−0.0175 (14)
C11	0.114 (2)	0.0604 (16)	0.098 (2)	−0.0283 (15)	−0.0061 (18)	−0.0100 (15)
C12	0.0861 (19)	0.0638 (16)	0.0819 (18)	−0.0268 (13)	−0.0108 (14)	0.0053 (13)
C13	0.109 (3)	0.089 (2)	0.068 (2)	−0.020 (2)	−0.008 (2)	−0.0094 (17)
O1	0.1232 (16)	0.0742 (12)	0.0787 (13)	−0.0397 (11)	−0.0029 (11)	0.0077 (9)
O2	0.1157 (16)	0.0851 (13)	0.0658 (12)	−0.0288 (11)	−0.0009 (10)	0.0022 (9)
N1	0.0818 (14)	0.0638 (12)	0.0560 (12)	−0.0253 (10)	−0.0094 (10)	0.0037 (9)
N2	0.0982 (16)	0.0621 (12)	0.0620 (13)	−0.0322 (11)	−0.0071 (11)	0.0015 (10)
S1	0.1117 (6)	0.0647 (4)	0.0625 (4)	−0.0339 (4)	−0.0004 (4)	−0.0005 (3)
F2	0.136 (8)	0.138 (5)	0.089 (5)	0.021 (5)	0.031 (4)	0.016 (4)
F1	0.163 (4)	0.182 (10)	0.086 (5)	−0.060 (4)	−0.054 (3)	0.026 (5)
F3	0.207 (8)	0.123 (7)	0.074 (3)	−0.091 (6)	0.020 (3)	−0.015 (3)
F11	0.41 (3)	0.196 (16)	0.067 (6)	−0.151 (18)	−0.075 (12)	0.003 (7)
F21	0.113 (8)	0.227 (14)	0.118 (12)	−0.063 (8)	0.011 (7)	0.006 (8)
F31	0.217 (12)	0.073 (7)	0.106 (8)	0.011 (6)	0.045 (6)	0.038 (5)
C1A	0.0672 (16)	0.0751 (16)	0.0594 (14)	−0.0291 (13)	−0.0031 (12)	0.0023 (12)
C2A	0.0668 (15)	0.0703 (15)	0.0507 (13)	−0.0261 (12)	−0.0040 (11)	−0.0008 (11)
C3A	0.0679 (16)	0.0687 (15)	0.0607 (15)	−0.0226 (12)	−0.0024 (12)	0.0025 (12)
C4A	0.0832 (19)	0.0805 (17)	0.0715 (17)	−0.0252 (14)	0.0075 (14)	0.0086 (14)
C5A	0.103 (2)	0.094 (2)	0.0589 (16)	−0.0181 (17)	0.0139 (15)	0.0060 (15)
C6A	0.110 (2)	0.091 (2)	0.0592 (17)	−0.0194 (17)	0.0034 (15)	−0.0131 (14)
C7A	0.0622 (14)	0.0672 (15)	0.0573 (14)	−0.0245 (11)	−0.0042 (11)	−0.0004 (11)
C8A	0.0800 (17)	0.0635 (15)	0.0594 (14)	−0.0247 (13)	−0.0023 (12)	−0.0035 (11)
C9A	0.0635 (15)	0.0742 (16)	0.0630 (15)	−0.0253 (12)	−0.0045 (12)	−0.0073 (12)
C10A	0.0750 (18)	0.0735 (17)	0.0778 (18)	−0.0154 (14)	−0.0004 (14)	−0.0146 (14)
C11A	0.0845 (19)	0.0595 (15)	0.101 (2)	−0.0181 (14)	−0.0119 (16)	−0.0042 (15)
C12A	0.0759 (17)	0.0647 (16)	0.0743 (17)	−0.0261 (13)	−0.0091 (13)	0.0072 (13)
C13A	0.091 (2)	0.095 (2)	0.0635 (19)	−0.031 (2)	0.0036 (17)	−0.0125 (17)
O1A	0.1020 (14)	0.0825 (12)	0.0745 (12)	−0.0487 (11)	0.0130 (10)	−0.0082 (9)
O2A	0.0917 (13)	0.0776 (11)	0.0596 (10)	−0.0294 (9)	0.0022 (9)	−0.0031 (8)
N1A	0.0781 (13)	0.0646 (12)	0.0553 (11)	−0.0304 (10)	0.0014 (10)	−0.0011 (9)
N2A	0.0778 (13)	0.0687 (13)	0.0573 (12)	−0.0334 (10)	0.0050 (10)	−0.0022 (10)
S1A	0.1139 (6)	0.0870 (5)	0.0643 (4)	−0.0586 (4)	0.0135 (4)	−0.0097 (3)
F2A	0.133 (4)	0.177 (9)	0.094 (4)	−0.046 (6)	−0.026 (4)	0.039 (5)
F3A	0.149 (5)	0.118 (4)	0.096 (6)	−0.033 (4)	0.059 (5)	−0.031 (4)
F1A	0.197 (9)	0.189 (8)	0.072 (2)	−0.150 (7)	−0.003 (5)	−0.005 (4)
F2B	0.178 (14)	0.104 (6)	0.094 (7)	0.020 (7)	0.056 (7)	0.040 (5)
F1B	0.106 (6)	0.248 (17)	0.164 (11)	−0.091 (8)	−0.016 (5)	0.084 (11)
F3B	0.35 (2)	0.20 (2)	0.059 (5)	−0.14 (2)	−0.011 (11)	−0.030 (7)

### Geometric parameters (Å, °)

**Table:**

C1—O1	1.224 (3)	C1A—O1A	1.223 (3)
C1—N1	1.376 (3)	C1A—N1A	1.376 (3)
C1—C3	1.448 (3)	C1A—C3A	1.449 (3)
C2—N2	1.334 (3)	C2A—N2A	1.336 (3)
C2—N1	1.391 (3)	C2A—N1A	1.390 (3)
C2—S1	1.649 (2)	C2A—S1A	1.648 (2)
C3—C4	1.333 (3)	C3A—C4A	1.344 (3)
C3—O2	1.355 (3)	C3A—O2A	1.365 (3)
C4—C5	1.412 (4)	C4A—C5A	1.401 (4)
C4—H4	0.93	C4A—H4A	0.93
C5—C6	1.312 (4)	C5A—C6A	1.328 (4)
C5—H5	0.93	C5A—H5A	0.93
C6—O2	1.354 (3)	C6A—O2A	1.357 (3)
C6—H6	0.93	C6A—H6A	0.93
C7—C12	1.379 (3)	C7A—C12A	1.378 (3)
C7—C8	1.388 (3)	C7A—C8A	1.387 (3)
C7—N2	1.408 (3)	C7A—N2A	1.409 (3)
C8—C9	1.372 (3)	C8A—C9A	1.377 (3)
C8—H8	0.93	C8A—H8A	0.93
C9—C10	1.380 (4)	C9A—C10A	1.379 (3)
C9—C13	1.490 (4)	C9A—C13A	1.487 (4)
C10—C11	1.357 (4)	C10A—C11A	1.374 (4)
C10—H10	0.93	C10A—H10A	0.93
C11—C12	1.371 (4)	C11A—C12A	1.368 (4)
C11—H11	0.93	C11A—H11A	0.93
C12—H12	0.93	C12A—H12A	0.93
C13—F2	1.299 (12)	C13A—F1A	1.281 (7)
C13—F1	1.303 (11)	C13A—F3A	1.296 (9)
C13—F3	1.322 (8)	C13A—F2A	1.335 (8)
N1—H1	0.86	N1A—H1A	0.86
N2—H2	0.86	N2A—H2A	0.86
			
O1—C1—N1	123.1 (2)	O1A—C1A—N1A	122.9 (2)
O1—C1—C3	121.0 (2)	O1A—C1A—C3A	120.9 (2)
N1—C1—C3	116.0 (2)	N1A—C1A—C3A	116.2 (2)
N2—C2—N1	114.0 (2)	N2A—C2A—N1A	114.75 (19)
N2—C2—S1	127.68 (18)	N2A—C2A—S1A	127.02 (17)
N1—C2—S1	118.35 (17)	N1A—C2A—S1A	118.22 (18)
C4—C3—O2	109.7 (2)	C4A—C3A—O2A	109.9 (2)
C4—C3—C1	132.1 (3)	C4A—C3A—C1A	132.0 (2)
O2—C3—C1	118.2 (2)	O2A—C3A—C1A	118.1 (2)
C3—C4—C5	106.4 (3)	C3A—C4A—C5A	106.5 (2)
C3—C4—H4	126.8	C3A—C4A—H4A	126.8
C5—C4—H4	126.8	C5A—C4A—H4A	126.8
C6—C5—C4	107.0 (3)	C6A—C5A—C4A	107.2 (2)
C6—C5—H5	126.5	C6A—C5A—H5A	126.4
C4—C5—H5	126.5	C4A—C5A—H5A	126.4
C5—C6—O2	110.4 (3)	C5A—C6A—O2A	110.5 (3)
C5—C6—H6	124.8	C5A—C6A—H6A	124.8
O2—C6—H6	124.8	O2A—C6A—H6A	124.8
C12—C7—C8	119.0 (2)	C12A—C7A—C8A	119.2 (2)
C12—C7—N2	115.4 (2)	C12A—C7A—N2A	116.9 (2)
C8—C7—N2	125.5 (2)	C8A—C7A—N2A	123.9 (2)
C9—C8—C7	119.6 (2)	C9A—C8A—C7A	119.7 (2)
C9—C8—H8	120.2	C9A—C8A—H8A	120.2
C7—C8—H8	120.2	C7A—C8A—H8A	120.2
C8—C9—C10	120.8 (2)	C8A—C9A—C10A	120.8 (2)
C8—C9—C13	119.8 (2)	C8A—C9A—C13A	118.5 (2)
C10—C9—C13	119.4 (3)	C10A—C9A—C13A	120.7 (2)
C11—C10—C9	119.2 (3)	C11A—C10A—C9A	119.2 (2)
C11—C10—H10	120.4	C11A—C10A—H10A	120.4
C9—C10—H10	120.4	C9A—C10A—H10A	120.4
C10—C11—C12	120.9 (3)	C12A—C11A—C10A	120.5 (2)
C10—C11—H11	119.6	C12A—C11A—H11A	119.7
C12—C11—H11	119.6	C10A—C11A—H11A	119.7
C11—C12—C7	120.4 (3)	C11A—C12A—C7A	120.7 (2)
C11—C12—H12	119.8	C11A—C12A—H12A	119.6
C7—C12—H12	119.8	C7A—C12A—H12A	119.6
F2—C13—F1	103.4 (9)	F1A—C13A—F3A	107.4 (7)
F2—C13—F3	103.2 (9)	F1A—C13A—F2A	104.8 (6)
F1—C13—F3	104.9 (8)	F3A—C13A—F2A	102.3 (6)
F2—C13—C9	115.6 (5)	F1A—C13A—C9A	114.6 (4)
F1—C13—C9	114.0 (6)	F3A—C13A—C9A	114.2 (6)
F3—C13—C9	114.4 (5)	F2A—C13A—C9A	112.4 (4)
C6—O2—C3	106.6 (2)	C6A—O2A—C3A	106.0 (2)
C1—N1—C2	128.9 (2)	C1A—N1A—C2A	128.6 (2)
C1—N1—H1	115.6	C1A—N1A—H1A	115.7
C2—N1—H1	115.6	C2A—N1A—H1A	115.7
C2—N2—C7	132.2 (2)	C2A—N2A—C7A	130.37 (19)
C2—N2—H2	113.9	C2A—N2A—H2A	114.8
C7—N2—H2	113.9	C7A—N2A—H2A	114.8
			
O1—C1—C3—C4	0.4 (5)	O1A—C1A—C3A—C4A	0.3 (5)
N1—C1—C3—C4	−179.9 (3)	N1A—C1A—C3A—C4A	−178.7 (3)
O1—C1—C3—O2	180.0 (2)	O1A—C1A—C3A—O2A	178.2 (2)
N1—C1—C3—O2	−0.3 (3)	N1A—C1A—C3A—O2A	−0.8 (3)
O2—C3—C4—C5	0.9 (3)	O2A—C3A—C4A—C5A	−0.4 (3)
C1—C3—C4—C5	−179.4 (3)	C1A—C3A—C4A—C5A	177.6 (3)
C3—C4—C5—C6	−0.7 (4)	C3A—C4A—C5A—C6A	0.1 (4)
C4—C5—C6—O2	0.2 (4)	C4A—C5A—C6A—O2A	0.2 (4)
C12—C7—C8—C9	−0.8 (4)	C12A—C7A—C8A—C9A	−1.9 (4)
N2—C7—C8—C9	177.7 (2)	N2A—C7A—C8A—C9A	−179.7 (2)
C7—C8—C9—C10	0.7 (4)	C7A—C8A—C9A—C10A	1.6 (4)
C7—C8—C9—C13	−177.2 (3)	C7A—C8A—C9A—C13A	−178.1 (3)
C8—C9—C10—C11	0.1 (4)	C8A—C9A—C10A—C11A	−0.6 (4)
C13—C9—C10—C11	178.0 (3)	C13A—C9A—C10A—C11A	179.1 (3)
C9—C10—C11—C12	−0.8 (5)	C9A—C10A—C11A—C12A	0.1 (4)
C10—C11—C12—C7	0.7 (5)	C10A—C11A—C12A—C7A	−0.5 (4)
C8—C7—C12—C11	0.1 (4)	C8A—C7A—C12A—C11A	1.4 (4)
N2—C7—C12—C11	−178.5 (3)	N2A—C7A—C12A—C11A	179.3 (2)
C8—C9—C13—F2	−126.8 (9)	C8A—C9A—C13A—F1A	51.0 (9)
C10—C9—C13—F2	55.2 (10)	C10A—C9A—C13A—F1A	−128.7 (9)
C8—C9—C13—F1	113.5 (7)	C8A—C9A—C13A—F3A	175.5 (6)
C10—C9—C13—F1	−64.4 (8)	C10A—C9A—C13A—F3A	−4.2 (7)
C8—C9—C13—F3	−7.2 (7)	C8A—C9A—C13A—F2A	−68.5 (7)
C10—C9—C13—F3	174.9 (6)	C10A—C9A—C13A—F2A	111.8 (7)
C5—C6—O2—C3	0.4 (4)	C5A—C6A—O2A—C3A	−0.4 (3)
C4—C3—O2—C6	−0.8 (3)	C4A—C3A—O2A—C6A	0.5 (3)
C1—C3—O2—C6	179.4 (2)	C1A—C3A—O2A—C6A	−177.8 (2)
O1—C1—N1—C2	2.3 (4)	O1A—C1A—N1A—C2A	−11.4 (4)
C3—C1—N1—C2	−177.4 (2)	C3A—C1A—N1A—C2A	167.5 (2)
N2—C2—N1—C1	−5.4 (4)	N2A—C2A—N1A—C1A	7.6 (4)
S1—C2—N1—C1	174.32 (19)	S1A—C2A—N1A—C1A	−171.20 (19)
N1—C2—N2—C7	−177.3 (2)	N1A—C2A—N2A—C7A	−175.4 (2)
S1—C2—N2—C7	3.0 (4)	S1A—C2A—N2A—C7A	3.3 (4)
C12—C7—N2—C2	176.1 (3)	C12A—C7A—N2A—C2A	153.4 (2)
C8—C7—N2—C2	−2.4 (4)	C8A—C7A—N2A—C2A	−28.8 (4)

### Hydrogen-bond geometry (Å, °)

**Table:**

*D*—H···*A*	*D*—H	H···*A*	*D*···*A*	*D*—H···*A*
N1—H1···S1A^i^	0.86	2.78	3.643 (2)	176
N1—H1···O2	0.86	2.27	2.692 (3)	110
N1A—H1A···S1^ii^	0.86	2.74	3.592 (2)	173
N1A—H1A···O2A	0.86	2.30	2.700 (3)	108
N2—H2···O1	0.86	1.88	2.625 (3)	144
N2A—H2A···O1A	0.86	1.92	2.640 (3)	140

Symmetry codes: (i) *x*, *y*−1, *z*; (ii) *x*, *y*+1, *z*.
